# Transthoracic Needle Biopsy of Pulmonary Nodules: Meteorological Conditions and the Risk of Pneumothorax and Chest Tube Placement

**DOI:** 10.3390/jcm8050727

**Published:** 2019-05-22

**Authors:** Bedros Taslakian, Varshaa Koneru, James S. Babb, Divya Sridhar

**Affiliations:** 1 Department of Radiology, Division of Vascular and Interventional Radiology, NYU Langone School of Medicine, New York, NY 10016, USA; James.Babb@nyumc.org (J.S.B.); divya.sridhar.md@gmail.com (D.S.); 2 Gandhi Medical College and Hospital, Secunderabad 500003, India; varshaak@gmail.com

**Keywords:** biopsy, pneumothorax, weather, lung

## Abstract

The purpose of this paper is to evaluate whether meteorological variables influence rates of pneumothorax and chest tube placement after percutaneous transthoracic needle biopsy (PTNB) of pulmonary nodules. A retrospective review of 338 consecutive PTNBs of pulmonary nodules at a single institution was performed. All procedures implemented a coaxial approach, using a 19-gauge outer guide needle for access and a 20-gauge core biopsy gun with or without a small-gauge aspiration needle for tissue sampling. Correlation between age, sex, smoking history, lesion size, meteorological variables, and frequency of complications were evaluated. Fisher exact, trend and *t* tests were used to evaluate the relationship between each factor and rates of pneumothorax and chest tube placement. A *p* value of less than 0.05 was considered to indicate a statistically significant difference. Pneumothorax occurred in 115 of 338 patients (34%). Chest tube placement was required in 30 patients (8.9%). No significant relationship was found between pneumothorax rate and age (*p* = 0.172), sex (*p* = 0.909), smoking history (*p* = 0.819), or lesion location (*p* = 0.765). The presence or absence of special weather conditions did not correlate with the rate of pneumothorax (*p* = 0.241) or chest tube placement (*p* = 0.213). The mean atmospheric temperature (*p* = 0.619) and degree of humidity (*p* = 0.858) also did not correlate with differences in the rate of pneumothorax. Finally, mean atmospheric pressure on the day of the procedure demonstrated no correlation with the rate of pneumothorax (*p* = 0.277) or chest tube placement (*p* = 0.767). In conclusion, no correlation is demonstrated between the occurrence of pneumothorax after PTNB of pulmonary nodules and the studied meteorological variables.

## 1. Introduction

Computed tomography (CT)-guided percutaneous transthoracic needle biopsy (PTNB) is a well-established, safe, effective, and commonly used procedure in the diagnosis of pulmonary lesions [1,2]. Pneumothorax remains the most frequent complication of PTNB, with a reported incidence ranging from 12 to 45% [3,4,5,6,7,8]. Tube thoracostomy is required for the treatment of some large, expanding, or symptomatic pneumothoraces after PTNB, with a reported rate ranging from 2 to 15% [3,4,5,6,7,8]. Patient management after pneumothorax typically requires prolonged observation, multiple imaging studies, and sometimes needle evacuation of air, while tube placement may necessitate admission for overnight observation. Thus, these complications significantly impact the ease, cost, and patient experience of PTNB of lung nodules, which is typically performed on an outpatient basis. The identification of risk factors may help predict, and therefore potentially avert, the development of these complications.

Previous studies have identified several risk factors for pneumothorax after PTNB of lung nodules, including a larger needle size [4], the presence of emphysema along the needle path [9,10], a lack of history of ipsilateral lung surgery [3,11], a greater number of needle passes [3,6,7], traversing interlobar fissures, and a shallow pleural puncture angle [7,11]. Other more controversial risk factors relate to patient age, sex, location and size of the target lesion, as well as patient position after the biopsy [3,4,6,7,8,9,10,11,12], and are less consistently supported by the literature.

The effects of meteorological parameters such as changes in atmospheric pressure and temperature on the incidence of pneumothorax after PTNB have not been previously studied; however, exposure to rapid changes of environmental pressure (e.g., scuba diving or flying) can cause pneumothorax in healthy individuals [13,14,15]. Boyle’s gas law states that the volume of a gas is inversely related to atmospheric pressure and directly related to temperature [16]; thus, many practitioners advise patients to avoid air travel after PTNB based on the belief that altitude and atmospheric pressure changes may pose significant risks for patients with pneumothorax; it should be noted that this risk has not been proven, nor has the optimal time interval between pneumothorax resolution and safe travel by air been determined [17,18]. Studies have also observed that idiopathic spontaneous pneumothorax (ISP) cases occur in clusters [19,20,21,22], raising the possibility of an association with meteorological conditions; however, while the possible impact of changes in atmospheric pressure on the occurrence of ISP has been investigated [19,22,23,24,25,26], the results remain equivocal.

The aim of this study was to evaluate the possible associations of meteorological variables with pneumothorax development and need for chest tube placement following CT-guided PTNB of pulmonary nodules.

## 2. Materials and Methods

This study was approved by the institutional review board under waiver of informed consent and performed in compliance with the Health Insurance Portability and Accountability Act.

The electronic medical records of all patients who underwent CT-guided PTNB for focal lung lesions at a single institution between December 2011 and May 2014 were retrospectively reviewed. Each patient had a focal lung lesion that manifested as a nodule, a mass, or persistent infiltrate requiring pathologic diagnosis.

The following data were collected: patient sex and age, smoking history, number of pack-years in smokers, lobar location of the lung lesion, largest diameter of the target lesion on pre-procedural CT scan, presence or absence of a pneumothorax on post-procedural imaging, and whether the patient required a pneumothorax-related intervention (chest tube placement). For each of the 911 days of the study period, meteorological data were obtained from the local Central Park station, Manhattan, New York of the National Oceanic and Atmospheric Administration, which is located at the same altitude and within 3 miles of the medical center. Humidity, the mean atmospheric pressure (P_atm_), as well as the mean (T_atm_), minimum (MinT_atm_), and maximum (MaxT_atm_) atmospheric temperatures were recorded on the day of the procedure. To evaluate the impact of atmospheric pressure changes, the differences in the P_atm_ between the day of the procedure and one day prior (ΔP_atm_ − 1) and one day after (P_atm_ + 1) were also calculated. The presence or absence of special weather conditions (thunderstorms, rainfall, or snow) on the day of the procedure was noted.

Between December 2011 and May 2014, 368 CT-guided PTNB procedures for focal lung lesions were performed. Thirty procedures were excluded due to lack of complete weather data. A total of 338 patients were included in the final analysis. There were 166 (49%) male patients and 172 (51%) female patients, with an age range of 26–93 years (mean, 68 years; median, 70 years). Smoking history was obtained from the medical record. Of the 338 patients, 99 patients denied a smoking history, and 203 admitted to habitual smoking. Smoking history was missing in 36 patients. Number of pack-years was reported in 171 patients; 124 (72%) patients reported a smoking history of >16 pack-years. Lesion locations were the upper lobe in 167 (49%) patients, the middle lobe or lingula in 26 (8%) patients, and the lower lobe in 145 (43%) patients. The P_atm_ across all procedure days was 29.88 ± 0.21-inch Hg (median = 29.88). There was minimal variation between the P_atm_ on the day of the procedure and day before with a mean difference of 0.14 ± 0.12-inch Hg (median = 0.12); similar small difference was found between the P_atm_ on the day of the procedure and day after with a mean difference of 0.15 ± 0.12-inch Hg (median = 0.11). The mean T_atm_ was 50.86 ± 16.88 degrees F and mean humidity was 61.24 ± 16.47% across the data set.

### 2.1. Procedure

All biopsies were performed by one of seven board-certified interventional radiologists or by radiologists in training under the supervision of an interventional radiologist, in accordance with the protocol of the Vascular and Interventional Radiology Section at our institution. The interventional radiologists had 3 to 30 years of experience with CT-guided procedures. Patients were positioned to optimize access to the target lesion, and standard intermittent CT technique was used for imaging guidance. A 19-gauge guide needle was used for access in all cases. Specimens were then obtained with a 20-gauge core needle biopsy device (Bard^®^ Mission^®^ Disposable Core Biopsy Instrument; Bard Peripheral Vascular, Inc., Tempe, AZ, USA) in all cases. In some cases, fine needle aspiration was performed for cytology prior to core biopsy using a 21-gauge needle. Blood patches were not performed in any of the procedures per protocol at this institution.

After the procedure, CT images were obtained to check for immediate pneumothorax and other complications. Pneumothorax was defined as any visible retraction of the pleural surface from the parietal pleura detected on CT scans or chest radiographs. The patients were monitored in a holding unit after the procedure, and upright chest radiographs with posterior–anterior inspiratory and expiratory views were performed 1 to 2 h after the procedure. Additional radiographs were obtained if clinically warranted or for follow up of imaging abnormalities. If a small asymptomatic pneumothorax developed, the patient was treated conservatively with monitoring of vital signs, administration of supplemental oxygen, and follow-up chest radiography 1 h later to evaluate the stability of the pneumothorax. A chest tube was inserted if the pneumothorax was large (lung surface retraction of >2 cm), expanding, or symptomatic [27].

### 2.2. Statistical Analysis

Continuous variables were summarized as mean ± standard deviation, and categoric measures were summarized with frequency counts and percentages. Fisher exact tests were used to assess the association of pneumothorax and chest tube insertion with smoking history, nodule location, and special weather conditions. The Jonckheere–Terpstra (JT) trend test was used to assess the likelihood of each outcome to monotonically increase or decrease as a function of smoking history (pack-years). Unequal variance *t*-test was used to compare presence or absence of pneumothorax or chest tube insertion in terms of weather data. All statistical tests were conducted at the two-sided 5% significance level using SAS 9.3 (SAS Institute, Cary, NC, USA).

## 3. Results

Of the 338 PTNBs, 115 (34%) were complicated by pneumothorax. Chest tube placement was required in 30 patients; 26% of patients with pneumothorax and 8.9% of all patients undergoing biopsy. Multiple independent risk factors for pneumothorax were examined (Table 1).

Whether or not a patient admitted smoking did not significantly affect the pneumothorax (*p* = 0.898) or chest tube placement (*p* = 0.819) rates. Pneumothorax occurred in 71/203 (35%) patients with a smoking history compared to 36/99 (36%) patients without a smoking history. Chest tube placement was required in 18/71 (25%) of smokers who had pneumothorax compared to 10/36 (28%) of non-smokers who had pneumothorax. There was also no significant association between reported number of pack-years in smokers and either pneumothorax (*p* = 0.556) or chest tube insertion rates (*p* = 0.541).

No significant relationship was found between pneumothorax rate and age (*p* = 0.172), sex (*p* = 0.909), or lesion location (*p* = 0.765). The mean age of patients with pneumothorax was 69.6 ± 13.3, and the mean age of those without pneumothorax was 67.5 ± 12.9 (*p* = 0.172). When patients were divided into two age groups, 29/94 (31%) of patients younger than 62 years had pneumothorax compared to 86/244 (35%) of patients 62 years and older (*p* = 0.172). Of 166 male patients, 57 (34%) had pneumothorax compared to 58 of 172 (34%) female patients (*p* = 0.909). The Mean lesion size was slightly but significantly smaller in patients with pneumothorax than in patients without pneumothorax (2.44 ± 1.18 cm versus 2.95 ± 1.85 cm, *p* = 0.005).

The presence or absence of special weather conditions did not correlate with differences in the rate of pneumothorax or chest tube placement. Pneumothorax occurred in 80/215 (37%) of patients who underwent a PTNB on days without special weather conditions, in 32/109 (29%) of patients who were biopsied on rainy days, and in 3/14 (21%) of patients who underwent a biopsy on days with snow (*p* = 0.241) (Table 2).

A chest tube was required in 19/80 (24%) of patients who had a pneumothorax on days without special weather conditions, in 9/32 (28%) who had a pneumothorax on days with rainfall, and in 2/3 (67%) of patients who had a pneumothorax on days with snowfall (*p* = 0.213) (Table 3).

Neither the P_atm_ on the day of the procedure nor atmospheric pressure changes correlated significantly with the occurrence of pneumothorax. The P_atm_ on the day of the procedure in patients with pneumothorax was 29.89 ± 0.23 inch Hg compared to 29.87 ± 0.20 inch Hg in patients without pneumothorax (*p* = 0.277). There was also no significant correlation between the T_atm_ or degree of humidity and the rate of pneumothorax. The mean T_atm_ was 58.83 ± 16.52 degrees F in patients with pneumothorax and 57.87 ± 17.39 degrees F in patients without pneumothorax (*p* = 0.619). The mean humidity was 61.03 ± 17.40% in patient with pneumothorax compared to 61.39 ± 16.02% in patients without pneumothorax (*p* = 0.858) (Table 4).

The P_atm_ on the day of the procedure and atmospheric pressure variations also did not correlate with differences in the rates of chest tube placement. The P_atm_ on the day of the procedure in patients with pneumothorax who required a chest tube placement was 29.90 ± 0.21 inch Hg compared to 29.89 ± 0.24 inch Hg in patients with pneumothorax who did not require chest tube placement (*p* = 0.767). There was also no significant correlation between the T_atm_ or degree of humidity with the rate of chest tube placement in patients with pneumothorax. The mean T_atm_ was 58.97 ± 17.50 degrees F in patients with pneumothorax who required chest tube placement and 58.91 ± 16.22 degrees F in patients with pneumothorax who did not require chest tube placement (*p* = 0.987). The mean humidity was 61.50 ± 19.26% in patient with pneumothorax who required chest tube placement compared to 60.86 ± 16.80% in patients with pneumothorax who did not required chest tube placement (*p* = 0.876) (Table 5).

## 4. Discusssion

Pneumothorax remains the most common complication of CT-guided PTNB of pulmonary nodules, despite the use of contemporary best-practice techniques informed by studies of risk factors for this complication. In our study, the rate of pneumothorax was 34%, and that of chest tube placement was 8.9%, which are similar to rates described in the literature [3,4,5,6,7,8].

To our knowledge, this is the first study in the literature that evaluates the relationship between meteorological variables and the occurrence of complications after CT-guided PTNB of pulmonary nodules. We did not identify any evidence of biometeorological influence on the occurrence of pneumothorax or need for chest tube placement in patients undergoing CT-guided PTNB. Prior reports have observed that patients with ISP are hospitalized in clusters and have suggested that the occurrence of these pneumothoraces is therefore not a random event but indeed a meteoropathy [19,20,22]. Known predisposing conditions for pneumothorax, even in healthy individuals, include those in which rapid changes in environmental pressure lead to substantial transpulmonary pressure gradient, such as scuba diving [13] and commercial air travel [14]. In comparison, changes in P_atm_ of a few millimeters of mercury would seem excessively small as to induce pneumothoraces. However, small pressure variations of other origins (i.e., loud music) have also been reported as possible causes of spontaneous pneumothorax [28]. Even in studies confirming the more frequent occurrence of pneumothoraces with climatic variations, these differences are not so clear and remain controversial [19,22,23,24,26,29,30,31,32]. In our study, the average differences in the P_atm_ between the day of the procedure and day before and after the procedure were minimal. These changes are small compared to the changes in the P_atm_ during air travel. Atmospheric pressure and PiO2 are significantly lower at a typical aircraft cruising altitude of 40,000 feet than they are at sea level [33]. At 40,000 feet (12,192 m), the actual atmospheric pressure is 141 mmHg (5.5 inch Hg) whereas pressurization of the cabin on commercial airliners maintains cabin atmospheric pressures around 565 mmHg (22.2 inch Hg) (at sea level, atmospheric pressure is 760 mmHg (29.9 inch Hg)), which one would experience at an altitude of 8000 feet, called the “cabin altitude”. Boyle’s law states that the volume of a gas is inversely proportional to the pressure to which it is exposed. Thus, as barometric pressure falls in the aircraft cabin during the ascent, trapped air in any non-communicating body cavity (e.g., non-communicating pneumothorax, lung bleb, lung bulla, lung cyst, paranasal sinuses) will expand. It is estimated that the volume of air in a non-communicating body cavity, such as a pneumothorax, will increase by approximately 38 percent upon ascent from sea level to the maximum “cabin altitude” of 8000 feet (2438 m) [34,35]. Therefore, the presence of a pneumothorax at the time of a flight has been considered an absolute contraindication to air travel, and we still advise not travelling until complete resolution of a pneumothorax has been documented. However, the possibility that selected patients with a small, stable pneumothorax may be able to travel safely for a short flight was examined in an observational study of 50 patients with a pneumothorax following transthoracic needle aspiration biopsy [36]. Air travel within four days of a chest radiograph showing a residual pneumothorax was not associated with any need for in-flight medical attention and less than 8% of patients studied experienced minor chest symptoms during air travel. Further study is needed to evaluate the safety of short-duration air travel for patients with small, stable pneumothoraces, but without other underlying lung diseases.

Temperature has an influence on pressure (Boyle-Gay Lussac’s law), and therefore both should be measured simultaneously. However, temperature itself has not been reported to be a risk factor for developing pneumothorax since air temperature is equilibrated to body temperature by passing through the mouth or nose and the bronchial tract to the alveolar region under normal conditions.

Various studies have identified variables correlating with differences in the rate of pneumothorax after PTNB, including patient factors (age, sex, lung function, and presence of emphysema), lesion variables (size, depth, location, and pleural contact), and procedure-related factors (experience of the operator, degree of difficulty, imaging method used for guidance, and type/size of needle used) [3,4,5,6,7,8].

In the current study, there was no relationship demonstrated between patients’ smoking history and rate of pneumothorax or chest tube placement. These findings are similar to what Geraghty et al. found [4] and are in contrast to the widely held belief that smoking increases the risk of pneumothorax. Reports on this topic in the literature are mixed: some studies have shown no significant increase in pneumothorax in patients with abnormal pulmonary function tests [37], while others have demonstrated higher pneumothorax rates with obstructive lung disease [10,38,39].

There was no significant relationship shown in this study between the rates of pneumothorax and chest tube placement with patient sex, patient age, and lesion location in the lung. Although these have been described as risk factors for pneumothorax in some previous studies, available data in the literature on the association between pneumothorax rate and these variables are not consistent [4,6,7,8,9,10,11].

The main limitation of this study was the retrospective analysis of clinical records.

Second, some patients with late onset pneumothorax may have received treatment at other hospitals, and we did not have clinical data on these patients, which could influence the results. Third, there were no major meteorological events or changes in the study period (hurricane, tornado). Finally, meteorological parameters beyond pressure, temperature, and humidity (and combinations of these factors using multivariate analysis) were not assessed.

## 5. Conclusions

The results of the current study do not support the premise that the analyzed meteorological factors impact the occurrence of pneumothorax and the need for chest tube placement in patients undergoing CT-guided PTNB, suggesting that they are not the only risk factors for the onset of pneumothorax. Data correlating ISP to atmospheric pressure changes may not inform the discussion of post-biopsy pneumothorax, possibly due to mechanistic differences between the two conditions. We believe that the results of our study are significant, and further studies should focus on investigating other combinations of weather phenomena and potential triggering factors than airway pressure modifications, in order to elucidate the precise mechanisms of pneumothorax onset that might be modified in order to decrease their occurrence. We believe that more significant results could be obtained if similar studies are conducted using similar methods to establish comparisons between countries with different climates.

## Figures and Tables

**Table 1:** Risk factors of pneumothorax after computed tomography (CT)-guided lung biopsy.

Characteristic	Pneumothorax Absent	Pneumothorax Present	*p* Value
**Overall**	223 (66)	115 (34)	
Age *	67.5 ± 12.9	69.6 ± 13.3	0.172
**Age (y)**			0.522
<62	65 (69)	29 (31)	
≥62	158 (65)	86 (35)	
**Sex**			0.909
Male	109(66)	57 (34)	
Female	114 (66)	58 (34)	
**Smoking History**			0.898
Smoker	132 (65)	71 (35)	
Non-Smoker	63 (64)	36 (36)	
**Pack-Years**			0.556
<16	292 (62)	18 (38)	
16–29	21 (62)	13 (38)	
30–49	25 (66)	13 (34)	
>49	36 (69)	16 (31)	
**Lesion Size**			0.226
≤1.0 (cm)	16 (53)	14 (47)	
>1.0 (cm)	170 (66)	88 (34)	
**Lesion Size**			0.709
≤2.0 (cm)	76 (63)	44 (37)	
>2.0 (cm)	110 (65)	58 (35)	
**Lesion Location**			0.765
1. RUL	66 (65)	36 (35)	
2. RML	17 (85)	3 (15)	
3. RLL	42 (63)	25 (37)	
4. LUL	42 (65)	23 (35)	
5. Lingula	4 (67)	2 (33)	
6. LLL	53 (68)	25 (32)	

Note: Unless otherwise indicated, data are numbers of patients, and data in parentheses are percentages. * Data are mean ± standard deviation. RUL: right upper lobe; RML: right middle lobe; RLL: right lower lobe; LUL: left upper lobe; LLL: left lower lobe.

**Table 2:** Effect of the presence or absence of special weather conditions on the rate of pneumothorax after CT-guided lung biopsy.

Characteristic	Pneumothorax Absent	Pneumothorax Present
None	135/215 (63%)	80/215 (37%)
Thunderstorm	0 (0%)	0 (0%)
Rainfall	77/109 (71%)	32/109 (29%)
Snow	11/14 (79%)	3/14 (21%)

Note: Data are fractions of patients, percentages in parentheses. *p* = 0.241 (from the Fisher exact test of the association of rate of pneumothorax with the presence or absence of special weather conditions).

**Table 3:** Effect of the presence or absence of special weather conditions on the rate of chest tube placement after CT-guided lung biopsy.

Characteristic	Chest Tube Absent	Chest Tube Present
None	62/80 (76%)	19 (24)
Thunderstorm	0 (0%)	0 (0%)
Rainfall	23/32 (72%)	9/32 (28%)
Snow	1/3 (33%)	2/3 (67%)

Note: Data are fractions of patients, percentages in parentheses. *p* = 0.213 (from the Fisher exact test of the association of rate of chest tube placement with special weather conditions).

**Table 4:** Effect of the atmospheric variables on the rate of pneumothorax after CT-guided lung biopsy.

Atmospheric Variable	Pneumothorax Absent	Pneumothorax Present	*p*
**P_atm_** (inch Hg)	29.87 ± 0.20	29.89 ± 0.23	0.277
**ΔP_atm_ − 1** (inch Hg)	0.14 ± 0.12	0.15 ± 0.13	0.553
**ΔP_atm_ + 1** (inch Hg)	0.15 ± 0.13	0.13 ± 0.12	0.211
**T_atm_** (degrees F)	57.87 ± 17.39	58.83 ± 16.52	0.619
**MaxT_atm_** (degrees F)	65.31 ± 17.88	66.21 ± 17.25	0.654
**MinT_atm_** (degrees F)	50.55 ± 17.23	51.46 ± 16.23	0.632
**Humidity** (%)	61.39 ± 16.02	61.03 ± 17.40	0.858

Note: Data are mean ± standard deviation. *p* values are from the unequal variance *t* test.

**Table 5:** Effect of the atmospheric variables on the rate of chest tube placement after CT-guided lung biopsy.

Atmospheric Variable	Chest Tube Absent	Chest Tube Present	*p*
**P_atm_** (inch Hg)	29.89 ± 0.24	29.90 ± 0.21	0.767
**ΔP_atm_ − 1** (inch Hg)	0.15 ± 0.13	0.15 ± 0.11	0.985
**ΔP_atm_ + 1** (inch Hg)	0.13 ± 0.11	0.13 ± 0.15	0.913
**T_atm_** (degrees F)	58.91 ± 16.22	58.97 ± 17.50	0.987
**MaxT_atm_** (degrees F)	66.62 ± 16.75	65.57 ± 18.83	0.787
**MinT_atm_** (degrees F)	51.21 ± 16.17	52.43 ± 16.44	0.725
**Humidity** (%)	60.86 ± 16.80	61.50 ± 19.26	0.876

Note: Data are mean ± standard deviation. *p* values are from the unequal variance *t* test.
